# Proton Pump Inhibitors: The Culprit for Barrett’s Esophagus?

**DOI:** 10.3389/fonc.2014.00373

**Published:** 2015-01-09

**Authors:** Omran Alsalahi, Anca D. Dobrian

**Affiliations:** ^1^Department of Physiological Sciences, Eastern Virginia Medical School, Norfolk, VA, USA

**Keywords:** bile, proton pump inhibitors, Barrett’s esophagus, esophageal cancer, adenocarcinoma, NSAID, *Helicobacter pylori*

## Introduction

Barrett’s esophagus (BE) is a condition in which the stratified squamous epithelium (SSE) of the distal esophagus undergoes intestinal metaplasia (transformation to columnar epithelium), which predisposes the epithelium to esophageal adenocarcinoma (EAC) (1). The etiologic consensus for BE, remains a matter of debate; however, strong association with chronic gastroesophageal reflux disease (GERD) has been documented (2). An accurate representation of the prevalence for BE is still not clear, most likely due to a lack of protocol for screening (3). The alarming increase of EAC by 600% for the past 25 years suggests that BE has increased as well, as the latter represents the main risk factor for EAC (4–6). This emphasizes the importance of better understanding the causal process leading to intestinal metaplasia (BE) and suggests that a possible re-evaluation of the current protocol for the management and treatment of GERD and BE may be beneficial.

## Etiologic Hypothesis: PPIs Transiently Increase Intra-Gastric pH Leading to Bile Salt Toxicity

Originally, it was believed that chronic acid reflux was responsible for BE, as most patients who develop intestinal metaplasia have GERD. However, this may not be the case, as the increased use of proton pump inhibitors (PPIs) – introduced in the late 1980s (7) – appears to be associated with the increased incidence of EAC (8, 9). For example, a recent nationwide case–control study in Denmark showed that chronic long-term use of PPIs was associated with a significant increase in the risk of developing EAC in patients with BE (10). Thus, it is possible that chronic PPI use might promote the metaplasia (BE)-dysplasia-carcinoma (EAC) sequence (8, 11); however, a mechanistic explanation of the proposed scenario is currently missing. We hypothesize that (i) a temporally sustained albeit transient increase in the gastric pH, may cause bile salts to become soluble in the proximity of the lower esophageal sphincter (LES) where they may mobilize to the esophageal tract during reflux episodes, and (ii) during a short event of failed acid suppression in the esophagus, protonated bile salts may diffuse into the epithelial cells causing the mucosal metaplasia that could lead to BE.

## Bile Salts vs. Bile Acids: Which are Likely the More Potent Inducers of BE in the PPI Treated GERD?

The mechanism(s) by which bile and various bile acids (BA) may cause intestinal metaplasia has yet to be elucidated. While bile has been shown to reduce squamous differentiation in primary esophageal cell lines (12, 13), one of the major questions remains which of the BA/salts are the more potent inducers of epithelial metaplasia in the esophagus *in vivo*? Bile salts are formed in the liver by conjugating BA – cholic acid (CA) and chenodeoxycholic acid (CDCA) – with taurine (pKa 2) and glycine (pKa 3.7) to form tauroconjugates (TC) and glycoconjugates (GC), respectively. The physiological consequences of the lower pKa of the bile salts are that by ionizing in the small intestine (pH of 6–8) (14), they have better emulsifying properties and remain in the intestinal lumen due to their negative charge. *In vitro* evidence suggests that the secondary BA, deoxycholic acid, and lithocholic acid, are more potent inducers of intestinal metaplasia (9, 15), since they are more lipophilic and readily diffuse across the cell membranes. However, both the site of formation and the physico-chemical properties of the secondary BA make them unlikely candidates *in vivo*. First, secondary BA are formed by intestinal microbiota in the terminal ileum and the anaerobic bacteria in the colon (11), which are distal to the foregut and require a neutral pH environment. Second, secondary BA have poor solubility, and their inability to ionize at the gastric pH, largely prevents them from reaching the esophagus in sufficient quantities to induce metaplasia.

Interestingly, one of the *in vitro* mechanisms by which bile has been shown to reduce squamous differentiation in primary esophageal cell lines was via transcriptional up-regulation of the caudal-type homeobox proteins, Cdx1 and Cdx2. These transcription factors are known to promote the proliferation and differentiation of intestinal epithelial cells (12, 13). Furthermore, Cdx1/2 have been shown to activate transcription of the apical sodium-dependent bile acid transporter (ASBT) in BE (16, 17). ASBT is expressed in the ileum and has a major role in bile salt reabsorption. This mechanistic evidence leads us once more to believe that conjugated BA (bile salts), rather than secondary BA, are more likely to induce intestinal metaplasia. Our hypothesis is further supported by the relatively low pKa of bile salts compared to that of BA, which makes the former readily ionized in the context of the transiently increased intra-gastric pH environment of patients with GERD and treated with PPIs. Finally, a significant number of patients with BE are overweight (18, 19), and EAC has the strongest known association with body mass index (BMI) (20, 21). Therefore, obese patients may be exposed to higher levels of bile salts vs. BA, as the production of the former is dominant in response to high-lipid intake (98% bile salts, vs. <2% BA) (22). Importantly, it has been shown that patients with reflux disease have higher concentration of conjugated BA in their esophageal aspirates (23), especially during the postprandial periods (24).

## Steps Leading to Bile Salts Access to Esophageal Epithelium

### Step 1: Transiently high-gastric pH induced by long-term PPI treatment

Studies have shown that dose escalation of PPIs improves intra-gastric pH control (25, 26). The effectiveness of PPIs in controlling acid-related symptoms has resulted in their widespread use (27). However, in such an environment, the majority of bile salts, most likely GCs, potentially, may ionize and mobilize upstream into the esophagus. Thus, patients on long-term PPI treatment, and with a dysfunctional LES, may be at increased risk for BE and EAC. This hypothesis may also explain why GERD patients on PPIs, with a long history of severe reflux/heartburn (secondary to low-LES pressure), develop “long-segment” BE (>3 cm); while patients with a short history of heartburn (higher LES pressure), develop “short-segment” BE (<3 cm) (28, 29). Interestingly, in the former, the risk of EAC has been estimated to be 2–15 times higher (30). In terms of TC, the same concept applies; however, its ability to ascend to the esophagus would not require a higher gastric pH environment (due to low pKa = 1–2).

### Step 2: Lower than normal esophageal pH due to acid reflux facilitates diffusion of the bile salts in the epithelial cells

The second component of the pathogenesis that should be considered is the mechanism by which bile salts cross the esophageal membrane to promote epithelial de-differentiation and metaplasia. Patients with GERD regardless of how well they respond to PPI, still endure at least one reflux episode (intra-esophageal pH <4) per day. As such, trapped ionized GC bile salts may become protonated to a more hydrophobic state, thus, enhancing their ability to diffuse across the cell membrane (same concept applies for TC if pH is low enough). This idea is supported by studies that have shown that PPIs do not provide consistent acid suppression. Notably, in one study, it was reported that the majority of patients with long-segment BE who received different dosages of esomeprazole (Nexium), a second-generation PPI, had an intra-gastric pH >4 for 81–88% of the day (the higher the dosage the longer the duration) (26). Importantly, regardless of the dosage, during a 24-h monitoring period, up to 5% of the time for >75% of the patients (>5% for 16–23% of patients) the intra-esophageal pH was lower than 4.

**Figure 1:**
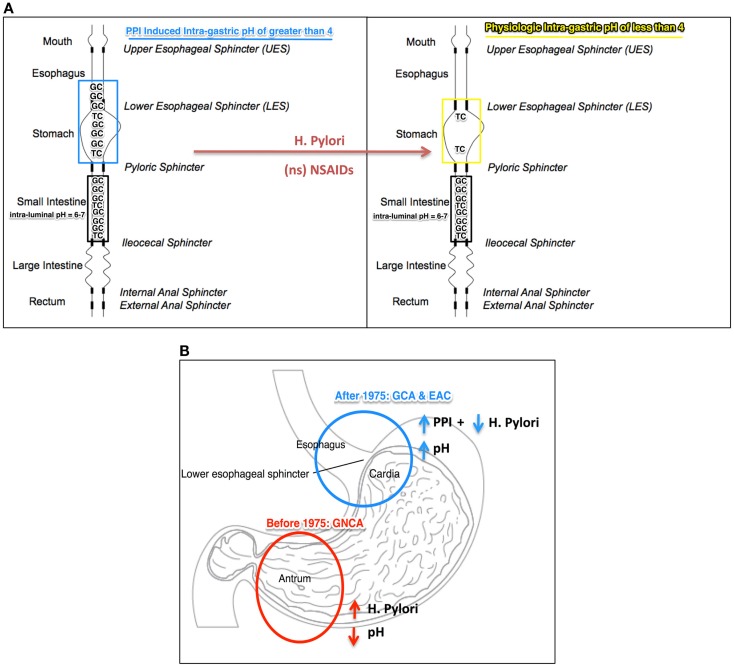
**(A)** Illustration of the “ion-trapping concept”: [intra-gastric pH (PPI induced or physiologic) = pKa (TC or GC) + log (Ionized TC or GC/Un-ionized TC or GC)] in which intra-gastric pH, PPI induced (blue) and physiological (yellow), facilitates movement of tauroconjugates (TC, pKa 2) and/or glycol-conjugates (GC, pKa 3.7) from the duodenum to the esophagus. When the intra-gastric pH is >4 (PPI induced), theoretically, 4 times more of the amount of ionized bile salts may mobilize to the esophagus. *Helicobacter pylori* (HP) and non-specific (ns) NSAID may increase acid secretion and shift the intra-gastric pH to lower than 4 (“safe-zone”), thereby preventing bile salt ionization. **(B)** Anatomical representation of the location of malignancy with high incidence rate in the United States, before and after 1975: non-cardia adenocarcinoma (GNCA), in red, before 1975 when *H. pylori* infection was high and PPI not in chronic use; gastric cardia adenocarcinoma (GCA) and esophageal adenocarcinoma (EAC), in blue, after 1975, with reduced incidence of *H. pylori* and the advent of long-term use of PPI. TG, tauroconjugate; GC, glycoconjugate.

Overall, we believe that the PPI-induced increase of the intra-gastric pH to >4 could promote higher levels of conjugated BA to reach the esophagus. During episodes of acid reflux, when the intra-esophageal pH transiently decreases to <4, conjugated BA may become protonated (hydrophobic) and therefore can cross the esophageal membrane. The “ion-trapping concept” (pH = pKa + log I−/U) explains this phenomenon: the higher than normal the intra-gastric pH, the greater the amount of ionized bile salts that will reach the esophagus; the lower than normal the intra-esophageal pH, the more bile salts in un-ionized form that may potentially cross the epithelial cell membrane (Figure 1A).

## *Helicobacter pylori*/NSAIDs Ensure Maintenance of an Intra-Gastric pH “Safe-Zone” Below 4 in the Context of PPI Treatment

*Helicobacter pylori* (HP) infection and non-specific NSAID have been associated with reduced incidences of esophageal intestinal metaplasia and adenocarcinoma. Furthermore, this has been observed for patients who had regularly taken acid-suppressing medication. The reasons behind this inverse association remain unknown. Congruent with our hypothesis, we propose that HP infection and/or NSAIDs may be countering the effect of acid-suppressing medications by establishing a steady intra-gastric pH lower than 4, which we believe is the “safe-zone” that may limit the reflux of ionized conjugated BAs (Figure 1B).

### NSAIDs

Frequent use of NSAIDs has been strongly associated with reduced incidence of neoplastic progression in patients with BE (31, 32). The inhibition of cyclooxygenase-2 (COX-2) – found to be elevated in epithelial cells of BE during the progression from low-grade to high-grade dysplasia (precursor to EAC) (33) – has been proposed as a possible chemoprotective mechanism (34). However, selective COX-2 inhibitors had no effect on the incidence of EAC (34–36). Interestingly, non-selective NSAID (nsNSAID) – especially aspirin (irreversible COX-1/2 inhibitor) – are strongly associated with decreased risk of EAC in patients with BE (37). Furthermore, this protective effect was also evident with the concomitant use of PPIs, demonstrating a longitudinal-response relationship – the longer the use, the lower the risk (34, 38).

Prostaglandins (PG), synthesized by cyclooxygenase enzymes, have been known to protect the gastric mucosa and to inhibit gastric acid secretion. Importantly, PGs derived from COX-1, but not COX-2, exert inhibitory effects on acid secretion (39). Thus, inhibition by non-specific NSAIDs may theoretically increase acid secretion in patients on PPI therapy, thereby countering the acid suppression effect of PPIs and promoting an intra-gastric pH 2–4. Further investigation is worth pursuing, in light of recent evidence demonstrating aspirin use is associated with risk reduction for BE in patients with GERD and on PPI therapy (40).

### *Helicobacter* *pylori*

*Helicobacter pylori* infection, in patients with GERD, has also been associated with decreased risk for BE in patients on anti-reflux medication (PPI or H2RA, at least once a week), and more protective for long-segment than short-segment BE (41). Increased gastric acidity ensued from HP infection, in subjects on anti-reflux medication, also, may maintain the intra-gastric pH safe-zone that we proposed to be relevant for preventing bile salts toxicity.

From a global health perspective, in Japan, the high-HP infection (CagA^+^ strains) may be causal for the lower frequency of BE (42). However, it should be noted that compared to the western world, Japan has a higher prevalence of gastric non-cardia adenocarcinoma (GNCA) – strongly correlated with CagA^+^ HP infection (43) – yet, low incidences of EAC (44). Furthermore, short-segment BE is more common in Japan, though increase in length is observed in older patients, while long-segment BE are more prevalent in western countries (45, 46). The reasons behind these epidemiological differences remain unknown. Nevertheless, the epidemiologic data raises the possibility that our hypothesis, supported by the ion-trapping concept and implying a role for the bile salts in the pathogenesis of BE and EA, may apply to the manifestation of gastric intestinal metaplasia (in gastric antrum) – a risk factor also strongly associated with GNCA and recently linked with bile (47).

Though gastric carcinogenesis is not directly addressed by the hypothesis discussed in this article, it is possible that the bile salts may have a mechanistic contribution considering the inverse association between the location of malignancy and the intra-gastric pH. High-acid secretion (pH 1–2), as rendered by CagA^+^ strains of HP, may promote bile salt (TG as the prime contributor) toxicity in the gastric antrum (more proximal to the duodenum); low-acid secretion (pH >4) as rendered by PPIs, may promote bile salt (GC, pKa 3.7, as the prime contributor) toxicity in the gastric cardia and lower esophagus (more distal to the duodenum). The “ion-trapping concept” may provide an explanation for HP’s (CagA^+^ strains) inverse association with adenocarcinomas of the upper stomach (gastric cardia carcinoma) and esophagus (EAC) (48, 49), and direct association with adenocarcinoma of the lower stomach (GNCA) (43, 48) (Figure 1B). Paralleling the decline in HP infections and the increased chronic use of PPIs, in the United States, since 1975, GNCA incidence rate was reduced while GCA has increased in conjecture with EAC occurrence (50).

## Significance

Bile has been shown to induce hyperplasia and metaplasia of the esophageal epithelium and therefore bile salts may be key contributors to BE and esophageal cancer. In this opinion article, we propose that an increase in the gastric pH induced by prolonged use of PPIs may ionize and hence facilitate bile salts transport to the esophagus during GERD and their subsequent diffusion into the esophageal epithelial cells. Therefore, it may be clinically relevant to more tightly control the gastric pH in subjects with GERD chronically treated with PPIs, in particular, in obese subjects where the bile salt production is increased. One therapeutic approach to achieve the balance of the gastric pH below 4 could be the use of combined NSAIDs and PPI therapy.

## Conflict of Interest Statement

The authors declare that the research was conducted in the absence of any commercial or financial relationships that could be construed as a potential conflict of interest.

## References

[1] Spechler SJ Barrett’s esophagus. N Engl J Med (2002) 346:836–42 10.1056/NEJMcp012118 11893796

[2] Gilbert EW Luna RA Harrison VL Hunter JG Barrett’s esophagus: a review of the literature. J Gastrointest Surg (2011) 15(5):708–18 10.1007/s11605-011-1485-y 21461873

[3] Sami SS Ragunath K Iyer PG. Screening for Barrett’s esophagus and esophageal adenocarcinoma: rationale, recent progress, challenges and future directions. Clin Gastroenterol Hepatol (2014). 10.1016/j.cgh.2014.03.036 24887058 PMC4254386

[4] Pohl H Welch HG The role of overdiagnosis and reclassification in the marked increase of esophageal adenocarcinoma incidence. J Natl Cancer Inst (2005) 97(2):142–6 10.1093/jnci/dji181 15657344

[5] Brown LM Devesa SS Chow W-H. Incidence of adenocarcinoma of the esophagus among white Americans by sex, stage, and age. J Natl Cancer Inst (2008) 100(16):1184–7. 10.1093/jnci/djn211 18695138 PMC2518165

[6] Wang KK Sampliner RE Diagnosis, surveillance and therapy of Barrett’s esophagus. Am J Gastroenterol (2008) 103:788–97 10.1111/j.1572-0241.2008.01835.x 18341497

[7] Garnett WR. History of acid suppression: focus on the hospital setting. Pharmacotherapy (2003) 23(10P2):56S–60S. 10.1592/phco.23.13.56S.31932 14587958

[8] Nasr AO Dillon MF Conlon S Downey P Chen G Ireland A Acid suppression increases rates of Barrett’s esophagus and esophageal injury in the presence of duodenal reflux. Surgery (2012) 151(3):382–90. 10.1016/j.surg.2011.08.021 22019500

[9] Huo X Juergens S Zhang X Rezaei D Yu C Strauch ED Deoxycholic acid causes DNA damage while inducing apoptotic resistance through NF-κB activation in benign Barrett’s epithelial cells. Am J Physiol Gastrointest Liver Physiol (2011) 301(2):G278–86 10.1152/ajpgi.00092.2011 21636532 PMC3154602

[10] Hvid-Jensen F Pedersen L Funch-Jensen P Drewes AM Proton pump inhibitor use may not prevent high-grade dysplasia and oesophageal adenocarcinoma in Barrett’s oesophagus: a nationwide study of 9883 patients. Aliment Pharmacol Ther (2014) 39(9):984–91 10.1111/apt.12693 24617286

[11] Ridlon JM Kang D-J Hylemon PB. Bile salt biotransformations by human intestinal bacteria. J Lipid Res (2006) 47(2):241–59. 10.1194/jlr.R500013-JLR200 16299351

[12] Kazumori H Ishihara S Kinoshita Y. Roles of caudal-related homeobox gene Cdx1 in oesophageal epithelial cells in Barrett’s epithelium development. Gut (2009) 58(5):620–8. 10.1136/gut.2008.152975 19136512

[13] Ghatak S Reveiller M Toia L Ivanov A Godfrey TE Peters JH. Bile acid at low ph reduces squamous differentiation and activates EGFR signaling in esophageal squamous cells in 3-D culture. J Gastrointest Surg (2013) 17(10):1723–31. 10.1007/s11605-013-2287-1 23921815

[14] Hofmann AF Mysels KJ. Bile acid solubility and precipitation in vitro and in vivo: the role of conjugation, pH, and Ca2+ ions. J Lipid Res (1992) 33(5):617–26. 1619357

[15] Spechler SJ. Does Barrett’s esophagus regress after surgery (or proton pump inhibitors)? Dig Dis (2014) 32(1–2):156–63. 10.1159/000357184 24603402

[16] Zhao J Gregersen H. Relationships of CDXs and apical sodium-dependent bile acid transporter in Barrett’s esophagus. World J Gastroenterol (2013) 19(18):2736. 10.3748/wjg.v19.i18.2736 23687410 PMC3653147

[17] Ma L Jüttner M Kullak-Ublick GA Eloranta JJ. Regulation of the gene encoding the intestinal bile acid transporter ASBT by the caudal-type homeobox proteins CDX1 and CDX2. Am J Physiol Gastrointest Liver Physiol (2012) 302(1):G123–33. 10.1152/ajpgi.00102.2011 22016432

[18] Westhoff B Brotze S Weston A McElhinney C Cherian R Mayo MS The frequency of Barrett’s esophagus in high-risk patients with chronic GERD. Gastrointest Endosc (2005) 61(2):226–31. 10.1016/S0016-5107(04)02589-1 15729230

[19] El-Serag HB Kvapil P Hacken-Bitar J Kramer JR. Abdominal obesity and the risk of Barrett’s esophagus. Am J Gastroenterol (2005) 100(10):2151–6. 10.1111/j.1572-0241.2005.00251.x 16181362

[20] Renehan AG Tyson M Egger M Heller RF Zwahlen M. Body-mass index and incidence of cancer: a systematic review and meta-analysis of prospective observational studies. Lancet (2008) 371:569–78. 10.1016/S0140-6736(08)60269-X 18280327

[21] Lagergren J Influence of obesity on the risk of esophageal disorders. Nat Rev Gastroenterol Hepatol (2011) 8:340–7 10.1038/nrgastro.2011.73 21643038

[22] McQuaid KR Laine L Fennerty MB Souza R Spechler SJ. Systematic review: the role of bile acids in the pathogenesis of gastro-oesophageal reflux disease and related neoplasia. Aliment Pharmacol Ther (2011) 34(2):146–65. 10.1111/j.1365-2036.2011.04709.x 21615439

[23] Nehra D Howell P Williams CP Pye JK Beynon J. Toxic bile acids in gastro-oesophageal reflux disease: influence of gastric acidity. Gut (1999) 44(5):598–602. 10.1136/gut.44.5.598 10205192 PMC1727508

[24] Kauer WK Peters JH DeMeester TR Feussner H Ireland AP Stein HJ Composition and concentration of bile acid reflux into the esophagus of patients with gastroesophageal reflux disease. Surgery (1997) 122(5):874–81. 10.1016/S0039-6060(97)90327-5 9369886

[25] Castell DO Kahrilas PJ Richter JE Vakil NB Johnson DA Zuckerman S Esomeprazole (40 mg) compared with lansoprazole (30 mg) in the treatment of erosive esophagitis. Am J Gastroenterol (2002) 97(3):575–83. 10.1111/j.1572-0241.2002.05532.x 11922549

[26] Spechler SJ Sharma P Traxler B Levine D Falk GW. Gastric and esophageal pH in patients with Barrett’s esophagus treated with three esomeprazole dosages: a randomized, double-blind, crossover trial. Am J Gastroenterol (2006) 101(9):1964–71. 10.1111/j.1572-0241.2006.00661.x 16848802

[27] Weber HC. Proton pump inhibitor therapy and potential long-term harm. Curr Opin Endocrinol Diabetes Obes (2014) 21(1):1–2. 10.1097/MED.0000000000000031 24310148

[28] Sharma P Morales TG Sampliner RE. Short segment Barrett’s esophagus – the need for standardization of the definition and of endoscopic criteria. Am J Gastroenterol (1998) 93(7):1033–6. 10.1016/S0002-9270(98)00205-6 9672325

[29] Loughney T Maydonovitch CL Wong RKH. Esophageal manometry and ambulatory 24-hour pH monitoring in patients with short and long segment Barrett’s esophagus. Am J Gastroenterol (1998) 93(6):916–9. 10.1111/j.1572-0241.1998.00276.x 9647018

[30] Rudolph RE Vaughan TL Storer BE Haggitt RC Rabinovitch PS Levine DS Effect of segment length on risk for neoplastic progression in patients with Barrett esophagus. Ann Intern Med (2000) 132(8):612–20. 10.7326/0003-4819-132-8-200004180-00003 10766679

[31] Vaughan TL Dong LM Blount PL Ayub K Odze RD Sanchez CA Non-steroidal anti-inflammatory drugs and risk of neoplastic progression in Barrett’s oesophagus: a prospective study. Lancet Oncol (2005) 6(12):945–52. 10.1016/S1470-2045(05)70431-9 16321762

[32] Tsibouris P Hendrickse MT Isaacs PET. Daily use of non-steroidal anti-inflammatory drugs is less frequent in patients with Barrett’s oesophagus who develop an oesophageal adenocarcinoma. Aliment Pharmacol Ther (2004) 20(6):645–55. 10.1111/j.1365-2036.2004.02150.x 15352913

[33] Morris CD Armstrong GR Bigley G Green H Attwood SE. Cyclooxygenase-2 expression in the Barrett’s metaplasia-dysplasia-adenocarcinoma sequence. Am J Gastroenterol (2001) 96(4):990–6. 10.1016/S0002-9270(00)02392-3 11316217

[34] Kastelein F Spaander MC Biermann K Steyerberg EW Kuipers EJ Bruno MJ Nonsteroidal anti-inflammatory drugs and statins have chemopreventative effects in patients with Barrett’s esophagus. Gastroenterology (2011) 141(6):2000–8. 10.1053/j.gastro.2011.08.036 21878200

[35] Róka R Inczefi O Kiss P Gyetvai Á Bálint L Németh I The effect of combined long term aspirin and proton pump inhibitor therapy on the histological progression of Barrett’s metaplasia. Z Gastroenterol (2014) 52(05):A55 10.1055/s-0034-1376115

[36] Heath EI Canto MI Piantadosi S Montgomery E Weinstein WM Herman JG Secondary chemoprevention of Barrett’s esophagus with celecoxib: results of a randomized trial. J Natl Cancer Inst (2007) 99(7):545–57. 10.1093/jnci/djk112 17405999 PMC3755596

[37] Tsibouris P Vlachou E Isaacs PET. Role of chemoprophylaxis with either NSAIDs or statins in patients with Barrett’s esophagus. World J Gastrointest Pharmacol Ther (2014) 5(1):27–39. 10.4292/wjgpt.v5.i1.27 24605249 PMC3944467

[38] Nguyen DM Richardson P El-Serag HB. Medications (NSAIDs, statins, proton pump inhibitors) and the risk of esophageal adenocarcinoma in patients with Barrett’s esophagus. Gastroenterology (2010) 138(7):2260–6. 10.1053/j.gastro.2010.02.045 20188100 PMC2883678

[39] Barnett K Bell CJ McKnight W Dicay M Sharkey KA Wallace JL. Role of cyclooxygenase-2 in modulating gastric acid secretion in the normal and inflamed rat stomach. Am J Physiol Gastrointest Liver Physiol (2000) 279(6):G1292–7. 11093953 10.1152/ajpgi.2000.279.6.G1292

[40] Omer ZB Ananthakrishnan AN Nattinger KJ Cole EB Lin JJ Kong CY Aspirin protects against Barrett’s esophagus in a multivariate logistic regression analysis. Clin Gastroenterol Hepatol (2012) 10(7):722–7. 10.1016/j.cgh.2012.02.031 22426086 PMC3435144

[41] Fischbach LA Graham DY Kramer JR Rugge M Verstovsek G Parente P Association between *Helicobacter pylori* and Barrett’s esophagus: a case-control study. Am J Gastroenterol (2014) 109(3):357–68. 10.1038/ajg.2013.443 24419485 PMC4046944

[42] Fujita M Nakamura Y Kasashima S Furukawa M Misaka R Nagahara H. Risk factors associated with Barrett’s epithelial dysplasia. World J Gastroenterol (2014) 20(15):4353. 10.3748/wjg.v20.i15.4353 24764673 PMC3989971

[43] Huang JQ Zheng GF Sumanac K Irvine EJ Hunt RH. Meta-analysis of the relationship between cagA seropositivity and gastric cancer. Gastroenterology (2003) 125:1636–44. 10.1053/j.gastro.2003.08.033 14724815

[44] Hongo M Nagasaki Y Shoji T. Epidemiology of esophageal cancer: orient to occident. Effects of chronology, geography and ethnicity. J Gastroenterol Hepatol (2009) 24(5):729–35. 10.1111/j.1440-1746.2009.05824.x 19646015

[45] Okita K Amano Y Takahashi Y Mishima Y Moriyama N Ishimura N Barrett’s esophagus in Japanese patients: its prevalence, form, and elongation. J Gastroenterol (2008) 43(12):928–34. 10.1007/s00535-008-2261-y 19107336

[46] Goh K-L. Gastroesophageal reflux disease in Asia: a historical perspective and present challenges. J Gastroenterol Hepatol (2011) 26(s1):2–10. 10.1111/j.1440-1746.2010.06534.x 21199509

[47] Tatsugami M Ito M Tanaka S Yoshihara M Matsui H Haruma K Bile acid promotes intestinal metaplasia and gastric carcinogenesis. Cancer Epidemiol Biomarkers Prev (2012) 21(11):2101–7. 10.1158/1055-9965.EPI-12-0730 23010643

[48] Chow W-H Blaser MJ Blot WJ Gammon MD Vaughan TL Risch HA An inverse relation between cagA+ strains of *Helicobacter pylori* infection and risk of esophageal and gastric cardia adenocarcinoma. Cancer Res (1998) 58(4):588–90. 9485003

[49] Wu AH Crabtree JE Bernstein L Hawtin P Cockburn M Tseng CC Role of *Helicobacter pylori* CagA+ strains and risk of adenocarcinoma of the stomach and esophagus. Int J Cancer (2003) 103(6):815–21. 10.1002/ijc.10887 12516104

[50] Devesa SS Fraumeni JF The rising incidence of gastric cardia cancer. J Natl Cancer Inst (1999) 91(9):747–9 10.1093/jnci/91.9.747 10328099

